# Up-regulation of GABA_B_ Receptor Signaling by Constitutive Assembly with the K^+^ Channel Tetramerization Domain-containing Protein 12 (KCTD12)*

**DOI:** 10.1074/jbc.M113.476770

**Published:** 2013-07-10

**Authors:** Klara Ivankova, Rostislav Turecek, Thorsten Fritzius, Riad Seddik, Laurent Prezeau, Laëtitia Comps-Agrar, Jean-Philippe Pin, Bernd Fakler, Valerie Besseyrias, Martin Gassmann, Bernhard Bettler

**Affiliations:** From the ‡ Department of Biomedicine, University of Basel, CH-4056 Basel, Switzerland,; the § Institut de Génomique Fonctionnelle, CNRS, UMR 5203, University of Montpellier 1 and 2, F-34094 Montpellier, France,; ¶ U661 INSERM, F-34094 Montpellier, France,; the ‖ Institute of Physiology II, University of Freiburg, 79104 Freiburg, Germany, and; the ** Center for Biological Signaling Studies (bioss), 79108 Freiburg, Germany

**Keywords:** Bioluminescence Resonance Energy Transfer (BRET), G Protein-coupled Receptors (GPCR), GABA Receptors, Protein Assembly, Trafficking, Gamma-Aminobutyric Acid, GABA-B, Potassium Channel Tetramerization Domain

## Abstract

GABA_B_ receptors are the G-protein coupled receptors (GPCRs) for GABA, the main inhibitory neurotransmitter in the central nervous system. Native GABA_B_ receptors comprise principle and auxiliary subunits that regulate receptor properties in distinct ways. The principle subunits GABA_B1a_, GABA_B1b_, and GABA_B2_ form fully functional heteromeric GABA_B(1a,2)_ and GABA_B(1b,2)_ receptors. Principal subunits regulate forward trafficking of the receptors from the endoplasmic reticulum to the plasma membrane and control receptor distribution to axons and dendrites. The auxiliary subunits KCTD8, -12, -12b, and -16 are cytosolic proteins that influence agonist potency and G-protein signaling of GABA_B(1a,2)_ and GABA_B(1b,2)_ receptors. Here, we used transfected cells to study assembly, surface trafficking, and internalization of GABA_B_ receptors in the presence of the KCTD12 subunit. Using bimolecular fluorescence complementation and metabolic labeling, we show that GABA_B_ receptors associate with KCTD12 while they reside in the endoplasmic reticulum. Glycosylation experiments support that association with KCTD12 does not influence maturation of the receptor complex. Immunoprecipitation and bioluminescence resonance energy transfer experiments demonstrate that KCTD12 remains associated with the receptor during receptor activity and receptor internalization from the cell surface. We further show that KCTD12 reduces constitutive receptor internalization and thereby increases the magnitude of receptor signaling at the cell surface. Accordingly, knock-out or knockdown of KCTD12 in cultured hippocampal neurons reduces the magnitude of the GABA_B_ receptor-mediated K^+^ current response. In summary, our experiments support that the up-regulation of functional GABA_B_ receptors at the neuronal plasma membrane is an additional physiological role of the auxiliary subunit KCTD12.

## Teaser

**Background:** KCTD12 regulates agonist potency and kinetics of GABA_B_ receptor signaling.

**Results:** KCTD12 stably associates with GABA_B_ receptors as early as in the endoplasmic reticulum and reduces constitutive receptor internalization from the cell surface.

**Conclusion:** KCTD12 increases GABA_B_ receptor surface expression and the magnitude of receptor signaling.

**Significance:** KCTD12 not only regulates agonist potency and kinetics but also the magnitude of GABA_B_ receptor signaling.

## Introduction

GABA_B_ receptors play an important role in regulating neuronal excitability in the mammalian central nervous system. Accordingly, GABA_B_ receptors have been implicated in a variety of neurological and psychiatric conditions, including epilepsy, anxiety, depression, schizophrenia, addiction, and pain (1, 2). Native GABA_B_ receptors are reported to comprise principal and auxiliary subunits that assemble into molecularly and functionally distinct receptor subtypes (3–6). The principal subunits GABA_B1a_, GABA_B1b_, and GABA_B2_ have a typical seven-transmembrane domain topology and form fully functional heteromeric GABA_B(1a,2)_ and GABA_B(1b,2)_ receptors that regulate Kir3-type K^+^ channels, voltage-gated Ca^2+^ channels, and adenylate cyclase. It is well established that newly synthesized principal subunits assemble into heteromeric GABA_B(1,2)_ receptors in the ER.^3^ The masking of the ER retention motif RSRR in GABA_B1_ by GABA_B2_ ensures that only GABA_B1_ subunits that have assembled with GABA_B2_ subunits are efficiently routed out of the ER to the cell surface (1, 7, 8). The subunit isoform GABA_B1a_ contains targeting motifs in its primary sequence that traffic receptors to axonal sites (3). In contrast, the subunit isoform GABA_B1b_ traffics receptors to the somatodendritic compartment (3). The auxiliary subunits KCTD8, -12, -12b, and -16 (named after their K^+^ channel tetramerization domain) form tetramers that bind to the C-terminal cytoplasmic domain of GABA_B2_, in which mutation of a tyrosine residue to alanine (GABA_B2_Y902A) completely abolishes KCTD binding (5). The KCTDs increase agonist potency at the receptor and regulate rise time and desensitization of receptor-mediated K^+^ and Ca^2+^ current responses (5). GABA_B_ receptors are examples of GPCRs that are composed of principal and auxiliary subunits. Other examples are members of the calcitonin receptor family that associate with receptor activity-modifying proteins (9).

Prototypical protein complexes that are regulated by auxiliary subunits are voltage-gated (10) and ligand-gated ion channels (11, 12). Auxiliary subunits of ion channels directly and stably interact with a pore-forming subunit and modulate channel properties and/or surface trafficking of the channel complex. Auxiliary subunits of ion channels normally assemble with pore-forming subunits in the ER. Assembly in the ER is also observed with the receptor activity-modifying proteins, which promote cell surface expression of the associated GPCRs (9). Little is known about the interactions of auxiliary and principal subunits during the lifecycle of GABA_B_ receptors. For example, it is unknown where in the biosynthetic pathway auxiliary KCTD subunits assemble with principal subunits and whether the KCTDs influence surface trafficking or internalization of receptors. Likewise, it still has to be addressed whether the KCTDs stably associate with the receptor, generally considered a criterion to qualify as an auxiliary receptor subunit (12). Here, we show that KCTD12 associates with GABA_B2_ while the receptor resides in the ER. Moreover, we show that KCTD12 up-regulates receptors at the cell surface, presumably by reducing constitutive receptor internalization. Finally, we found that KCTD12 remains associated with the receptor upon receptor activation and during receptor internalization. In summary, our data support that KCTD12 is a stably associated auxiliary GABA_B_ receptor subunit that increases the magnitude of receptor signaling at the neuronal membrane.

## EXPERIMENTAL PROCEDURES

### 

#### 

##### Plasmids, Cell Culture, and Transfection

pCI plasmids encoding Myc-GABA_B1_,HA-GABA_B2_, Myc-GABA_B2,_ HA-GABA_B2_Y902A, and FLAG-tagged KCTDs were described previously (5, 13). Kir3.1/3.2 concatemers were cloned into pcDNA3.1 (14), and pEGFP-N1 was from Clontech. The yellow fluorescent protein (YFP) fragments YFP1 and YFP2 (15), were inserted C-terminally into pCI-HA-GABA_B2_, pCI-HA-GABA_B2_Y902A, and pCI-FLAG-KCTD12. The plasmids encoding C-terminally tagged HA-GABA_B1_-YFP (16) and Myc-GABA_B2_ (16) were described earlier. *Renilla* luciferase (Rluc) was inserted N-terminally to full-length KCTD12 and KCTD10 together with an SGGGGSGGG peptide linker. COS-1 cells were maintained in DMEM (Invitrogen) supplemented with 10% FBS (PAA Laboratories) at 37 °C with 5% CO_2_. CHO-K1 cells stably expressing GABA_B1_ and GABA_B2_ subunits were cultured as described (17). Lipofectamine 2000 (Invitrogen) was used for transient transfection of COS-1 and CHO-K1 cells. In the electrophysiology experiments, transfected cells were identified using the green fluorescent protein (GFP). The amount of DNA in the transfections was kept constant by supplementing with empty pCI plasmid (Promega). Cultured hippocampal neurons were prepared as described (18).

##### Cell ELISA

For ELISA quantification of Myc-GABA_B1_ at the plasma membrane, cells were fixed with 4% paraformaldehyde (Sigma) in PBS. Cells were then blocked with 10% normal goat serum (Invitrogen) and incubated with mouse anti-Myc monoclonal antibodies (1:500, 9E10, Roche Applied Science) for 1 h at 4 °C and horseradish peroxidase-conjugated anti-mouse antibodies (1:1000, GE Healthcare) for 35 min at room temperature. Luminescence was quantified with SuperSignal West Femto substrate (Pierce) in a Wallac VICTOR2^TM^ 1420 counter (PerkinElmer). Total Myc-GABA_B1_ was quantified after permeabilization of the cell membrane with 0.25% Triton X-100 (Sigma). FLAG-tagged KCTDs were quantified in permeabilized cells using mouse anti-FLAG antibodies (1:500, Sigma).

##### Immunocytochemistry

Cells were grown on glass coverslips coated with 0.5 mg/ml collagen (Serva), fixed, and blocked as described above. Surface Myc-GABA_B1_ was labeled with mouse anti-Myc antibodies (1:100) for 3 h at 4 °C. Total expression of Myc-GABA_B1_ was determined in permeabilized cells using rabbit anti-GABA_B1_ antibodies (1:500, Clone 25 (19)). Cells were washed with PBS and stained with secondary antibodies (Alexa Fluor goat anti-mouse 488 and Alexa Fluor goat anti-rabbit 647, 1:500, Molecular Probes) for 1 h at room temperature. For visualization of HA-GABA_B2_-YFP1 and HA-GABA_B2_Y902A-YFP1 in the YFP protein fragment complementation assay, cells expressing HA-GABA_B2_-YFP1 or HA-GABA_B2_Y902A-YFP1 with FLAG-KCTD12-YFP2 were incubated with mouse anti-HA (1:500, Covance) and Alexa Fluor goat anti-mouse 647 (1:500, Molecular Probes) antibodies. The ER was stained using rabbit anti-calnexin (1:500, Sigma) and Alexa Fluor goat anti-rabbit 568 (1:500, Molecular Probes) antibodies. Coverslips were mounted with FluorSave^TM^ Reagent (Calbiochem), and images were captured with a Leica DMI 4000B confocal microscope using identical laser settings.

##### Metabolic [^35^S]Methionine Labeling

For immunoprecipitation of FLAG-KCTD12, COS-1 cells were plated onto collagen-coated 100-mm dishes and starved in methionine-free DMEM (Sigma) for 30 min at 37 °C. Cells were then incubated in 3 ml of 100 μCi of EXPRESS^35^S protein labeling mix (PerkinElmer) for 15 min at 37 °C. Cells were rinsed with ice-cold PBS and harvested in 0.4 ml of lysis buffer (150 mm NaCl, 10 mm Tris-HCl, 5 mm EDTA, 1.5% Nonidet P-40) supplemented with protease inhibitors (complete Mini, Roche Applied Science). After the addition of 30 μl of 50% protein A-agarose (Roche Applied Science) with 2 μl of mouse anti-FLAG antibodies (Sigma), the lysate was incubated overnight at 4 °C. Agarose beads were washed in radioimmune precipitation buffer (150 mm NaCl, 10 mm Tris-HCl, 5 mm EDTA, 1.0% Nonidet P-40, 0.1% SDS, 0.5% deoxycholate). Immunoprecipitated FLAG-KCTD12 and Myc-GABA_B2_ proteins were identified by cross-correlation of autoradiography with Western blots using rabbit anti-FLAG and rabbit anti-Myc antibodies.

##### Cell Surface Immunoprecipitation

COS-1 cells expressing GABA_B_ receptors comprising Myc-tagged GABA_B1_ subunits were blocked with PBS containing 1% BSA and incubated with mouse anti-Myc antibodies (1:200) on ice for 3 h. After several washes with PBS, receptors were activated with 100 μm baclofen for 15 min. Cells were lysed, and protein G-agarose (Roche Applied Science) was added to the lysates. Immunoprecipitated proteins were resolved by SDS-PAGE and detected on Western blots. Detection of actin with mouse antibodies (1:1000, C4, Millipore) confirmed that surface receptors were immunoprecipitated.

##### Deglycosylation Experiments

Total protein lysates of transfected COS-1 were denatured in 0.05% SDS and 50 mm β-mercaptoethanol for 5 min at 37 °C and then digested with endoglycosidase H (Endo H, Roche Applied Science) for 3 h at 37 °C in 0.05 m sodium citrate buffer, pH 5.5. Digestion of denatured total protein with *N*-glycosidase F (Roche Applied Science) was performed in 0.05 m Tris buffer, pH 8.0, supplemented with 1% Nonidet P-40 and 10 mm 1,10-phenanthroline. Digestions were terminated by adding protein loading buffer. Proteins were resolved by SDS-PAGE and Western blots carried out using rabbit anti-GABA_B1_ (1:3000), guinea pig anti-GABA_B2_ (1:2000), and mouse anti-FLAG (1:3000) antibodies.

##### BRET Measurements

COS-1 transiently transfected with plasmids encoding Rluc and/or YFP fusion proteins were seeded into 96-well microplates (Greiner Bio-One) for 24 h. The Rluc substrate coelenterazine h (NanoLight Technologies) and the GABA_B_ receptor agonist baclofen were added for 15 min at a final concentration of 5 and 100 μm, respectively. Luminescence and fluorescence signals were detected sequentially using an Infinite® F500 microplate reader (Tecan). The BRET ratio was calculated as the ratio of the light emitted by GABA_B1_-YFP (530–570 nm) over the light emitted by Rluc-KCTD12 or Rluc-KCTD10 (370–470 nm). The BRET ratio was adjusted by subtracting the ratios obtained when Rluc fusion proteins were expressed alone. Total YFP fluorescence was measured with an excitation filter at 485 nm and an emission filter at 535 nm and corrected for the fluorescence measured in cells expressing Rluc fusion proteins in isolation. The results were expressed in milliBRET units, corresponding to the BRET ratio values multiplied by 1000. Each data point was obtained using quadruplicate wells of cells. Data were analyzed using GraphPad Prism 5.0 software.

##### Biotinylation Assay

Constitutive internalization of GABA_B_ receptors in transiently transfected COS-1 cells was studied as described (20). For detection of cell surface proteins, hippocampal neurons on 35-mm plates were incubated with 1 mg/ml Sulfo-NHS-SS-Biotin (Pierce) in PBS for 30 min at 4 °C. After quenching the biotinylation reaction with glycine and rinsing of the cells with ice-cold PBS, cells were scrapped from the plates and lysed. Biotinylated surface proteins were purified using NeutrAvidin-agarose (Pierce), washed, and resuspended in protein loading buffer. Protein samples were resolved by SDS-PAGE and Western blots carried out with rabbit anti-GABA_B1_, rabbit anti-KCTD12 (1:1000 (5)), and mouse anti-β-tubulin (1:3000, Sigma) antibodies.

##### Knockdown Experiments

Lentiviral vectors containing the KCTD12 and control shRNA sequences 5′-GCTATTACCTCAAGTTCAA-3′ and 5′-GTATCTCTTCATAGCCTTAT-3′, respectively (5), were constructed as described (21, 22) and produced using the HIV-1 packaging vectors Δ8.2 and vesicular stomatitis virus-G, kindly provided by Oliver Schlüter (European Neuroscience Institute, Göttingen). Cultured hippocampal neurons were transduced with lentiviruses for 2 h at DIV 6–7.

##### Electrophysiology

Transfected CHO-K1 cells and cultured hippocampal neurons were plated onto Thermanox^TM^ plastic coverslips (Nunc) or poly-l-lysine-coated glass coverslips, respectively. Electrophysiological recordings were performed as described (5). A fast superfusion system for drug application was used (23). For recordings and data analysis, we used pClamp10 software (Molecular Devices).

##### Data Analysis

With exception of the BRET data, which are expressed as ± S.D., all data are presented as mean ± S.E. If not stated otherwise, Student's *t* test with GraphPad Prism 5.04 was used. The band intensity on Western blots was quantified on unsaturated images using luminescent image analyzer LAS-4000 (Bucher Biotec AG, Basel) and ImageJ version 1.43 software (National Institutes of Health).

## RESULTS

### 

#### 

##### KCTD12 Increases Cell Surface Expression of GABA_B_ Receptors

**FIGURE 1.**
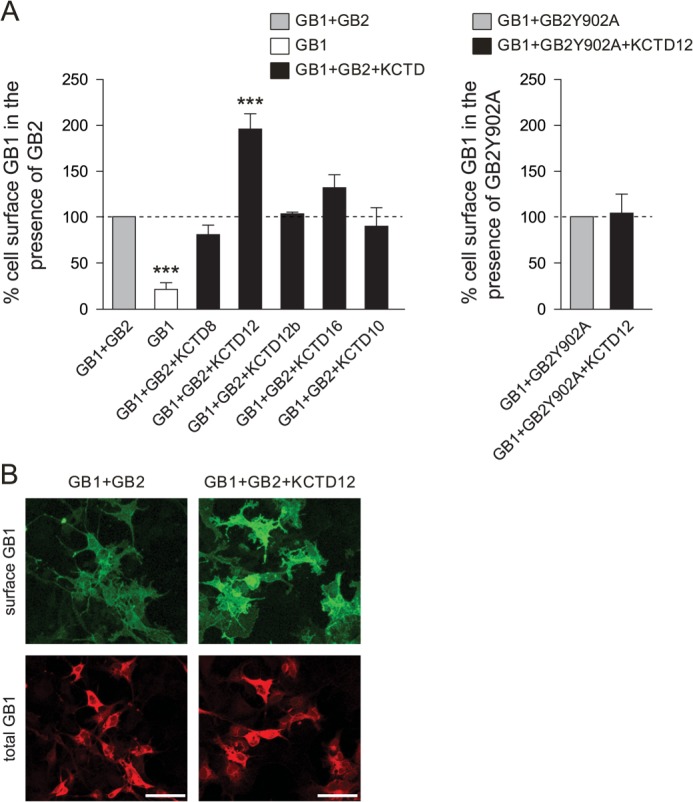
**KCTD12 promotes cell surface expression of GABA_B_ receptors in transfected COS-1 cells.** *A*, ELISA detection of Myc-GABA_B1_ (*GB1*)protein at the cell surface. Cells co-expressing GB1 and HA-GABA_B2_ (*GB2*) in the absence or presence of FLAG-tagged KCTDs (KCTD8, -12, -12b, -16, and -10) were labeled with anti-Myc antibodies for luminescence quantification of surface (nonpermeabilized cells) and total (permeabilized cells) GB1 protein. Selectively, KCTD12 promotes GB1 cell surface expression. KCTD10, which does not interact with GB2, had no effect on GB1 surface expression (*left*). KCTD12 did not promote GB1 surface expression when GB2 was replaced with HA-GABA_B2_Y902A (*GB2Y902A*) (5), a mutant GB2 subunit preventing KCTD12 binding (*right*). Surface GB1 protein was quantified by calculating the ratio of surface to total luminescence intensity. Values were normalized to control values in the absence of KCTDs (*GB1*+*GB2*). Separate control values were acquired for each experimental group. Data are from 4–7 independent experiments. ***, *p* < 0.001. *B*, immunofluorescence detection of GB1 protein at the cell surface. Cells co-expressing GB1 and GB2 in the absence and presence of KCTD12 were fluorescence-labeled with antibodies to image surface (anti-Myc, *green*) and total (anti-GABA_B1_, *red*) GB1 protein. Surface GB1 protein is significantly increased by 49.1 ± 9.1% (*p* < 0.001) in the presence of KCTD12. Maximum projection images were taken with a confocal microscope (*scale bar*, 100 μm).

We first analyzed whether the KCTDs influence GABA_B_ receptor surface expression. We expressed Myc-GABA_B1_ and HA-GABA_B2_ in the presence or absence of FLAG-tagged KCTD8, -12, -12b, and -16 in COS-1 cells. We used the extracellular Myc tag of GABA_B1_ to monitor with an ELISA the amount of GABA_B_ receptors at the cell surface. KCTD12 significantly promoted receptor surface expression (196.2 ± 16.5%, *p* < 0.001; Fig. 1*A*). In contrast, KCTD8, -12b, and -16 had no significant effect on receptor surface expression. As a control, KCTD10, a KCTD protein that does not bind to GABA_B_ receptors (5), had no effect on receptor surface expression either. Moreover, receptors without a KCTD-binding site (GABA_B2_Y902A (5)) exhibited no increase in surface expression in the presence of KCTD12 (Fig. 1*A*). Increased receptor surface expression therefore requires direct binding of KCTD12 to GABA_B2_. As expected, we observed low cell surface expression of GABA_B1_ in the absence of GABA_B2_ (21.4 ± 7.3% of the surface expression obtained in association with GABA_B2_, *p* < 0.001; Fig. 1*A*). Of note, GABA_B_ receptor surface expression in the absence as well as in the presence of KCTD12 was not significantly influenced by exposure to 100 μm baclofen for 15 min (data not shown), consistent with the reported lack of agonist-induced receptor internalization (20, 24–26).

We additionally examined receptor surface expression in the presence or absence of KCTD12 using confocal microscopy (Fig. 1*B*). Surface and total GABA_B1_ expression was monitored by immunolabeling prior (green fluorescence) and after (red fluorescence) permeabilization of the cells. In agreement with the ELISA data, the ratio of the GABA_B1_ surface fluorescence to the GABA_B1_ total fluorescence was significantly increased by 49.1 ± 9.1% (*p* < 0.001) in the presence of KCTD12.

##### KCTD12 Increases GABA_B_ Receptor-mediated Kir3 Current Amplitudes

**FIGURE 2.**
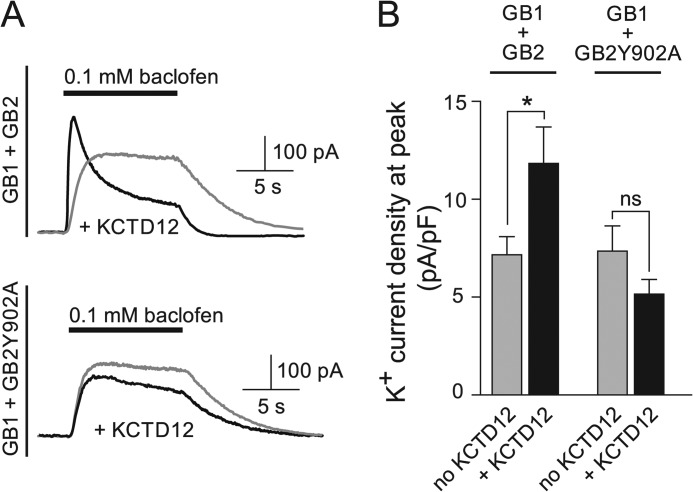
**KCTD12 increases the GABA_B_ receptor-mediated peak K^+^ current amplitude.** *A*, representative outward K^+^ currents evoked by fast application of baclofen to CHO-K1 cells expressing Kir3.1/3.2 channels and GABA_B_ receptors in the presence (*black traces*) or absence (*gray traces*) of KCTD12. GABA_B_ receptors were composed of either GABA_B1_ and GABA_B2_ (*GB1*+*GB2*) or GABA_B1_ and GABA_B2_Y902 (*GB1*+*GB2Y902A*) subunits. KCTD12 increases the peak K^+^ current at GB1+GB2 but not at GB1+GB2Y902A receptors. Whole-cell recordings were made at a holding potential of −50 mV. *B*, bar graph illustrating that KCTD12 enhances the K^+^ current density at the peak of the baclofen response in cells expressing GB1+GB2(GB1+GB2 + KCTD12, 11.8 ± 1.9 pA/pF; GB1+GB2, 7.12 ± 0.94 pA/pF; *, *p* < 0.05) but not in cells expressing GB1+GB2Y902A (GB1+GB2Y902A+KCTD12, 5.10 ± 0.78 pA/pF; GB1+GB2Y902A, 7.30 ± 1.31 pA/pF; *p* > 0.05). Each bar is the mean ± S.E. of 19–52 cells. *ns*, not significant.

We addressed whether increased GABA_B_ receptor surface expression in the presence of KCTD12 results in increased receptor-mediated Kir3 current amplitudes. We recorded outward K^+^ currents induced by fast application of the GABA_B_ receptor agonist baclofen (100 μm) to CHO-K1 cells expressing GABA_B_ receptors and Kir3.1/3.2 effector channels. In agreement with earlier data (5), we observed a shorter rise time and a pronounced rapid desensitization of baclofen-induced K^+^ currents in the presence of KCTD12 (Fig. 2*A*). Consistent with increased receptor surface expression in the presence of KCTD12, we observed a significantly increased K^+^ current density at the peak of the response (Fig. 2*B*). An increase in the peak K^+^ current density was not seen with receptors lacking the KCTD-binding site (GB1+GB2Y902A, Fig. 2*B*). Thus, electrophysiological experiments in heterologous cells support that binding of KCTD12 to GABA_B_ receptors increases the peak receptor response, consistent with the observed increase in receptor cell surface expression.

##### KCTD12 Assembles with GABA_B_ Receptors at the Cytoplasmic Side of the ER Membrane

**FIGURE 3.**
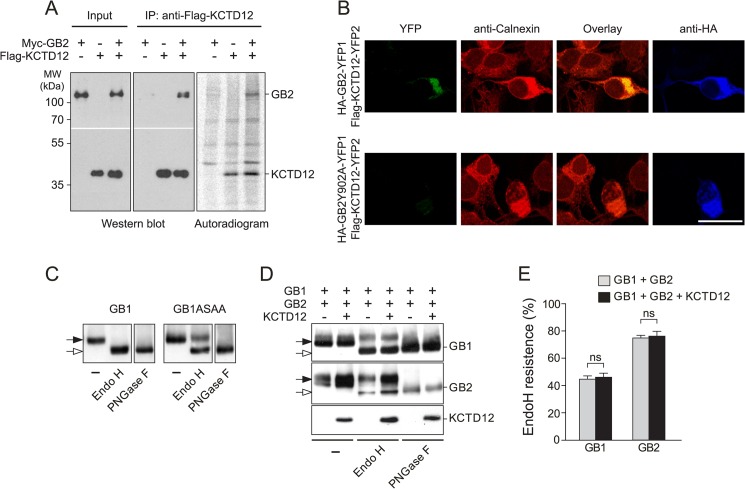
**KCTD12 assembles with GABA_B_ receptors at the cytoplasmic side of the ER membrane in transfected COS-1 cells.** *A*, cells co-expressing combinations of FLAG-KCTD12 and Myc-GABA_B2_ (*GB2*) were metabolically labeled with [^35^S]methionine for 15 min. After immunoprecipitation (*IP*) of FLAG-KCTD12, the precipitated ^35^S-labeled proteins were separated by SDS-PAGE and visualized using autoradiography. ^35^S-labeled Myc-GB2 and FLAG-KCTD12 proteins were identified by cross-correlation of autoradiographs with Western blots developed with anti-Myc and anti-FLAG antibodies, respectively. ^35^S-labeled Myc-GB2 protein co-precipitates with FLAG-KCTD12 protein, showing that KCTD12 interacts with newly synthesized GB2 residing in the ER. *MW*, molecular weight. *B*, a YFP protein fragment complementation assay between HA-GB2-YFP1 and FLAG-KCTD12-YFP2 was used to visualize the GABA_B2_-KCTD12 interaction in the secretory pathway. The fluorescence of reconstituted YFP overlapped with the ER marker calnexin, which was detected with anti-calnexin antibodies. As a negative control, expression of HA-GB2Y902A-YFP1, which does not bind to KCTD12, together with FLAG-KCTD12-YFP2 did not reconstitute YFP. Anti-HA antibodies were used to identify HA-GB2-YFP1 and HA-GB2Y902A-YFP1 in transfected cells (*scale bar*, 20 μm). *C*, Endo H and *N*-glycosidase F (*PNGase F*) treatment of Myc-GABA_B1_ (*GB1*) and Myc-GABA_B1_ASAA (*GB1ASAA*), a mutant GB1 protein that escapes ER retention. In contrast to GB1, the GB1ASAA protein is partially Endo H-resistant (*filled arrow*). *D*, glycosylation patterns of cell lysates expressing combinations of Myc-GABA_B1_ (*GB1*), HA-GABA_B2_ (*GB2*), and FLAG-KCTD12 (*KCTD12*) were analyzed on Western blots following Endo H or *N*-glycosidase F treatment. Endo H-resistant (*filled arrows*) and -sensitive (*open arrows*) forms of GB1 and GB2 are indicated. *E*, quantification of Endo H-resistant GB1 and GB2 protein did not reveal any significant alterations of subunit maturation in the presence of KCTD12. Data are the means ± S.E. of 3 independent experiments done in duplicates, *p* > 0.05. *ns*, not significant.

We next determined where in the biosynthetic pathway KCTD12 assembles with GABA_B_ receptors. We pulse-labeled COS-1 cells expressing GABA_B2_ and KCTD12 for 15 min with [^35^S]methionine, at which time newly synthesized transmembrane proteins have not yet progressed beyond the ER (27, 28). After 15 min of biosynthesis, immunoprecipitation with anti-FLAG antibodies not only purifies FLAG-KCTD12 but also efficiently co-precipitates ^35^S-labeled Myc-GABA_B2_ (Fig. 3*A*). This shows that FLAG-KCTD12 binds to newly synthesized ^35^S-labeled Myc-GABA_B2_ residing in the ER. The C termini of the GABA_B1_ and GABA_B2_ proteins are expected to face the cytoplasmic side of the ER membrane (7). Therefore KCTD12, a cytosolic protein, is assumed to assemble with GABA_B2_ at the cytoplasmic side of the ER membrane. We additionally studied assembly of KCTD12 with GABA_B_ receptors in living cells using a YFP protein fragment complementation assay (15). We fused the YFP1 and YFP2 fragments to the C termini of GABA_B2_ (GABA_B2_-YFP1) and KCTD12 (KCTD12-YFP2), respectively. Co-expression of GABA_B2_-YFP1 with KCTD12-YFP2 in COS-1 cells produced YFP fluorescence due to reconstitution of a functional YFP protein (Fig. 3*B*). The YFP fluorescence fully overlapped with the immunostaining pattern for the ER marker protein calnexin, showing that YFP reconstitution already takes place at the level of the ER (Fig. 3*B*). Binding of KCTD12-YFP2 to GABA_B2_-YFP1 was further confirmed in co-immunoprecipitation experiments (data not shown). In control experiments, we did not detect YFP fluorescence when expressing GABA_B2_-YFP1 or KCTD12-YFP2 in the absence of each other (data not shown). This indicates that the YFP fragments do not generate fluorescence on their own. Moreover, we did not observe YFP reconstitution when expressing GABA_B2_Y902A-YFP1, which does not bind to KCTD12, together with KCTD12-YFP2. Altogether, our data support that KCTD12 first assembles with GABA_B_ receptors at the cytoplasmic side of the ER membrane.

##### KCTD12 Does Not Measurably Influence Forward Trafficking of GABA_B_ Receptors

GABA_B_ receptor trafficking to the plasma membrane is regulated by sequence elements in the C termini of GABA_B1_ (for example the ER retention signal RSRR (1, 7, 8)) and GABA_B2_ (residues between amino acids 841 and 862 (29)). In fully assembled receptors, these sequence elements are located in proximity to Tyr-902 in GABA_B2_, the residue critically involved in KCTD binding (5). It is thus conceivable that binding of the KCTDs influences maturation and surface trafficking of GABA_B_ receptors. To monitor posttranslational modification in the biosynthetic pathway, we analyzed the glycosylation patterns of GABA_B1_ and GABA_B2_ in the presence and absence of KCTD12 in transfected COS-1 cells. Endo H removes *N*-linked mannose-rich oligosaccharides from proteins that reside in the ER. Because all later oligosaccharide structures during biosynthesis are resistant to Endo H, Endo H cleavage identifies the fraction of protein that resides in the ER. In addition, we used *N*-glycosidase F, which removes *N*-linked oligosaccharides from both mature and immature proteins, to demonstrate that in our experiments, all GABA_B_ receptors were accessible to the glycosidase treatment (Fig. 3, *C* and D). In the absence of GABA_B2_, all GABA_B1_ protein was Endo H-sensitive, in agreement with a lack of terminal GABA_B1_ glycosylation due to ER retention (Fig. 3*C*). In contrast, the Myc-GABA_B1_ASAA (GB1ASAA) protein, which escapes the ER because of mutation of the RSRR motif (13), was partially Endo H-resistant (Fig. 3*C*). Similarly, when GABA_B1_ was co-expressed with GABA_B2_, a fraction of GABA_B1_ protein became Endo H-resistant, showing that heteromeric assembly with GABA_B2_ promotes GABA_B1_ exit from the ER (Fig. 3*D*). We observed that more GABA_B2_ than GABA_B1_ protein was Endo H-resistant (GABA_B1_, 44.5 ± 2.4% of total GABA_B1_ protein; GABA_B2_, 74.6 ± 1.8% of total GABA_B2_ protein; Fig. 3*E*). This may be explained by limiting amounts of GABA_B2_ protein, which may only allow a fraction of GABA_B1_ protein to exit the ER. In addition, the lack of an ER retention signal in GABA_B2_ allows GABA_B2_ to efficiently exit the ER in the absence of GABA_B1_ (30, 31). Of importance for receptor maturation, we did not detect significant changes in the fraction of mature GABA_B1_ or GABA_B2_ protein in the presence of KCTD12 (GABA_B1_, 45.9 ± 3.1% of total GABA_B1_ protein; GABA_B2_, 76.2 ± 3.6% of total GABA_B2_ protein; *p* > 0.05 as compared with without KCTD12, nonparametric Mann-Whitney test; Fig. 3*E*). These biochemical data therefore support that maturation and forward trafficking of GABA_B_ receptors are not significantly altered in the presence of KCTD12.

##### KCTD12 Constitutively Associates with GABA_B_ Receptors

**FIGURE 4.**
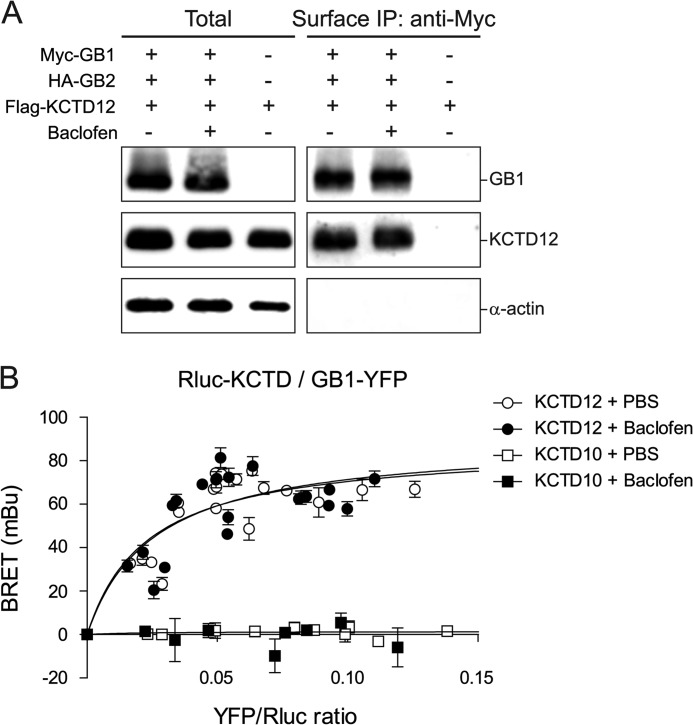
**KCTD12 stably interacts with GABA_B_ receptors during receptor activation in transfected COS-1 cells.** *A*, co-immunoprecipitation of KCTD12 with surface GABA_B_ receptors from cells expressing Myc-GABA_B1_ (*Myc-GB1*), HA-GABA_B2_ (*HA-GB2*), and FLAG-KCTD12 (*Flag-KCTD12*) with and without receptor activation with baclofen (100 μm, 15 min). The amount of KCTD12 protein that co-immunoprecipitated with GABA_B_ receptors was similar in the presence and absence of baclofen. The absence of α-actin in the immunoprecipitates (*IP*) supports that surface GABA_B_ receptors were selectively isolated. Data are representative of 3 independent experiments done in duplicates. *B*, BRET donor saturation curves were generated in cells expressing fixed amounts of Myc-GABA_B2_, Rluc-KCTD12, or Rluc-KCTD10 and increasing amounts of GABA_B1_-YFP (*GB1-YFP*). Cells were preincubated with 100 μm baclofen for 15 min (*filled circles and squares*) or with PBS only (*open circles and squares*). BRET is expressed as milliBRET units (*mBU*) determined as net *BRET* × 1000. The results are the mean ± S.D. of 3–4 individual saturation experiments. The curves were fitted using a nonlinear regression equation assuming a single binding site (GraphPad Prism).

Next we investigated whether the interaction of KCTD12 with GABA_B_ receptors at the cell surface is regulated by receptor activity. We used anti-Myc antibodies to immunoprecipitate GABA_B_ receptors from the cell surface of COS-1 cells expressing Myc-GABA_B1_, HA-GABA_B2_, and FLAG-KCTD12 (Fig. 4*A*). We co-immunoprecipitated equivalent amounts of KCTD12 with Myc-GABA_B1_ in the absence (PBS) and presence of baclofen (ratio of KCTD12:GABA_B1_ band intensity on Western blots; PBS, 0.84 ± 0.19; baclofen, 0.91 ± 0.11; *p* > 0.05, nonparametric Mann-Whitney test). These experiments show that KCTD12 is neither recruited nor dissociated from surface GABA_B_ receptors upon receptor activation.

We additionally studied KCTD12 assembly with GABA_B_ receptors in intact cells using BRET. We generated BRET donor saturation curves by co-expressing increasing amounts of GABA_B1_-YFP acceptor fusion protein with a fixed amount of Rluc-KCTD12 donor fusion protein (in the presence of a fixed amount of HA-GABA_B2_, Fig. 4*B*). The BRET signal shows a hyperbolic increase with increasing GABA_B1_-YFP expression (BRET_max_, 89.64 ± 5.37 milliBRET units; BRET_50_, 0.026 ± 0.005; Fig. 4*B*). This demonstrates a specific interaction of KCTD12 with GABA_B_ receptors (32). The BRET saturation curve was not altered in the presence of baclofen, showing that the fusion proteins remain associated during receptor activation (Fig. 4*B*). We only observed unspecific BRET signals when expressing increasing amounts of GABA_B1_-YFP with a fixed amount of Rluc-KCTD10, which does not interact with GABA_B_ receptors (in the presence of a fixed amount of HA-GABA_B2_; Fig. 4*B*). Biochemical and BRET experiments thus support that KCTD12 remains constitutively associated with the receptor during receptor activity.

##### Constitutive GABA_B_ Receptor Internalization Is Slowed in the Presence of KCTD12

**FIGURE 5.**
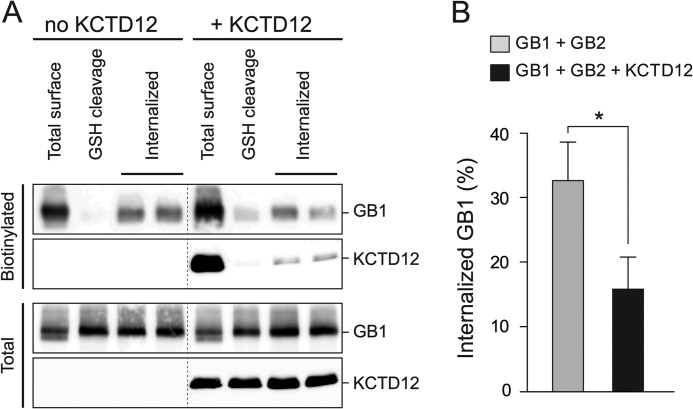
**KCTD12 reduces constitutive GABA_B_ receptor internalization in transfected COS-1 cells.** *A*, GABA_B_ receptor internalization in the presence or absence of KCTD12 was investigated using a biotinylation assay. Cell were biotinylated on ice for 15 min, washed, and then either incubated on ice for 120 min to prevent internalization of cell surface proteins (*Total surface*) or incubated at 37 °C for 120 min to allow internalization (*Internalized*). Biotinylated total surface and internalized proteins were purified with NeutrAvidin-Sepharose. GABA_B1_ (*GB1*) and KCTD12 protein was revealed on Western blots. For purification of biotinylated internalized protein, the biotin label at the cell surface was first removed by incubation with reduced glutathione (*GSH*). Biotinylated total surface protein cleaved by GSH (*GSH cleavage*) demonstrates that GSH efficiently removes the biotin label from surface proteins. Internalized GB1 and KCTD12 proteins (*Internalized*) were always analyzed in duplicates. *B*, quantification of GABA_B_ receptor internalization in the absence and presence of KCTD12. The percentage of the total GB1 protein that was internalized was calculated after subtraction of uncleaved GB1 protein after GSH cleavage at the cell surface. Bars are the means ± S.E. of 3 separate experiments. *, *p* < 0.05.

Because we did not observe significant changes in GABA_B_ receptor maturation and forward trafficking in the presence of KCTD12 (see above), we addressed whether KCTD12 possibly increases receptor surface levels by reducing constitutive endocytosis (20, 26, 33, 34). A cell surface biotinylation assay (20) indeed reveals a significantly reduced GABA_B_ receptor endocytosis in the presence of KCTD12 (Fig. 5*A*). Quantitative analysis showed that the percentage of total GABA_B1_ protein internalized within 120 min was significantly smaller in the presence of KCTD12 (KCTD12, 16 ± 5%; without KCTD12, 33 ± 6%; *p* < 0.05, nonparametric Mann-Whitney test; Fig. 5*B*). The rate of constitutive GABA_B_ receptor internalization in the absence of KCTD12 was similar to the rate reported in an earlier study (20). We conclude that KCTD12 increases cell surface GABA_B_ receptor expression by reducing constitutive receptor internalization. Co-immunoprecipitation experiments further reveal that GABA_B1_, GABA_B2_, and KCTD12 endocytose in associate with each other as a protein complex (Fig. 5*A*).

##### Acute and Constitutive Loss of KCTD12 in Cultured Hippocampal Neurons Reduces GABA_B_ Receptor Surface Levels

**FIGURE 6.**
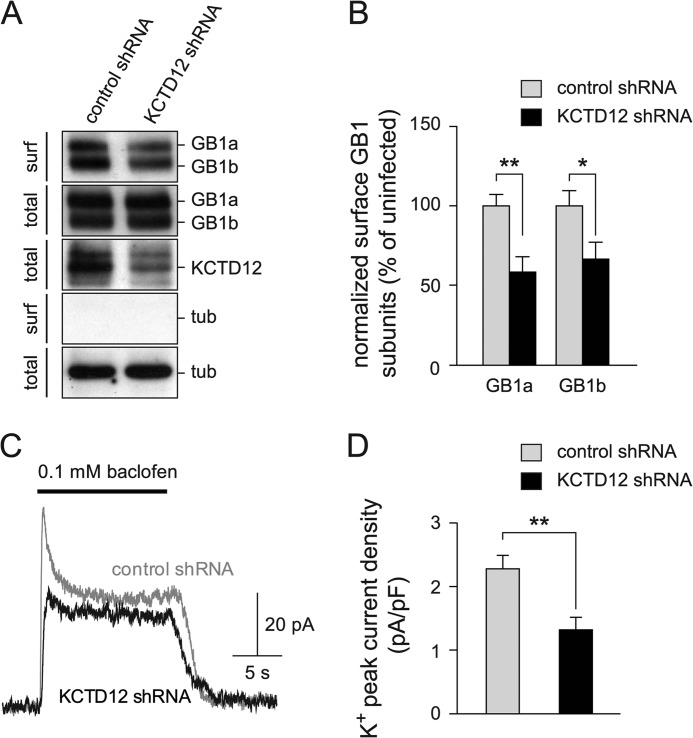
**Knockdown of KCTD12 reduces GABA_B_ receptor surface expression in hippocampal neurons.** *A*, cell surface biotinylation of mouse hippocampal neurons infected with KCTD12 shRNA or control shRNA. GABA_B1_ (*GB1a* and *GB1b*) and KCTD12 proteins in neuronal lysates (*total*) and NeutrAvidin-purified cell surface proteins (*surf*) were revealed on Western blots using specific antibodies. Tubulin (*tub*) was visualized to control for loading of the SDS-PAGE. *B*, graph illustrating the decrease of surface GB1a and GB1b protein in neurons after KCTD12 knockdown. Data are means ± S.E. from 5–8 experiments. *, *p* < 0.05, **, *p* < 0.01. *C*, representative GABA_B_ receptor-mediated K^+^ current responses recorded at −50 mV from cultured hippocampal neurons infected with control (*gray trace*) or KCTD12 shRNA (*black trace*). *D*, the bar graph shows that the K^+^ current density at the peak of the response is significantly reduced in neurons infected with KCTD12 shRNA as compared with neurons infected with control shRNA. Data are the means ± S.E., *n* = 10–17, **, *p* < 0.01.

**FIGURE 7.**
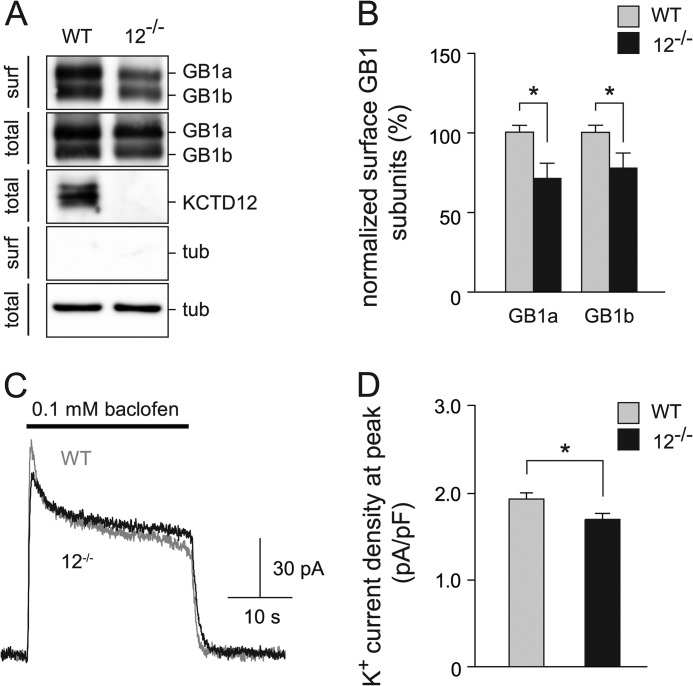
**Surface expression of GABA_B_ receptors is decreased in hippocampal neurons of *Kctd12*^−/−^ mice.** *A*, cell surface biotinylation assay with DIV 13 hippocampal neurons of *Kctd12*^−/−^ and control littermate mice. GABA_B1_ (*GB1a* and *GB1b*) and KCTD12 proteins in neuronal lysates (*total*) and NeutrAvidin-purified cell surface proteins (*surf*) were revealed on Western blots using specific antibodies. Tubulin (*tub*) was visualized to control for loading of the SDS-PAGE. *B*, graph illustrating the decrease of surface GB1a and GB1b protein in neurons of *Kctd12*^−/−^ mice. Data are means ± S.E. from 6–8 experiments. *, *p* < 0.05. *C*, representative GABA_B_ receptor-mediated K^+^ current responses evoked by baclofen application for 25 s to cultured hippocampal neurons of *Kctd12*^−/−^ (*black trace*) and control littermate mice (*gray trace*). Holding potential −50 mV. *D*, bar graph shows the K^+^ current density at peak of the response significantly reduced in *Kctd12*^−/−^ as compared with control neurons. Data are the means ± S.E., *n* = 14, *, *p* < 0.05.

Hippocampal neurons of mice express KCTD12 and KCTD16 protein (5, 6). We used a cell surface biotinylation assay to address whether down-regulation of endogenous KCTD12 protein in cultured hippocampal neurons influences GABA_B_ receptor expression at the plasma membrane. Neurons were infected at DIV 6–7 with lentiviruses encoding either KCTD12 shRNA or control shRNA and analyzed at DIV 13. KCTD12 shRNA significantly reduced the expression of endogenous KCTD12 protein as compared with control shRNA (55.8 ± 8.1% of control, KCTD12 protein was normalized to β-tubulin protein, *p* < 0.05; Fig. 6*A*). KCTD12 knockdown with shRNA resulted in a significant decrease of surface GABA_B1_ protein as compared with control shRNA (GABA_B1a_, 58.4 ± 9.7% of control, *p* < 0.01; GABA_B1b_, 66.4 ± 10.9%, *p* < 0.05; Fig. 6, *A* and *B*). In agreement with the results obtained from the cell surface biotinylation experiments, we found that knockdown of KCTD12 in hippocampal neurons significantly decreases the baclofen-induced K^+^ current density at the peak of the response (KCTD12 shRNA, 1.32 ± 0.20 pA/pF; control shRNA, 2.27 ± 0.21 pA/pF; *p* < 0.01; Fig. 6, *C* and *D*). Finally, we examined cell surface expression of GABA_B_ receptors in cultured hippocampal neurons of *Kctd12*^−/−^ mice (6). The cell surface biotinylation assay demonstrated that *Kctd12*^−/−^ neurons express reduced amounts of GABA_B1a_ and GABA_B1b_ proteins at the plasma membrane in comparison with wild-type neurons (GABA_B1a_, 71.5 ± 9.6% of WT; GABA_B1b_, 77.9 ± 9.4% of WT, *p* < 0.05; Fig. 7, *A* and *B*). We additionally tested GABA_B_ responses in *Kctd12*^−/−^ and wild-type neurons. The GABA_B_ receptor-mediated K^+^ current density at the peak of the response was slightly but significantly decreased in *Kctd12*^−/−^ as compared with wild-type neurons (*Kctd12*^−/−^, 1.70 ± 0.07 pA/pF; WT, 1.94 ± 0.07 pA/pF; *p* < 0.05; Fig. 7, *C* and *D*). Based on these electrophysiological results, we conclude that the presence of KCTD12 increases the maximal GABA_B_ receptor response in cultured hippocampal neurons.

## DISCUSSION

Over the past decade, the concept of principal and auxiliary subunits has been extended from voltage-gated ion channels to ligand-gated ion channels (11, 12) and GPCRs (9). Signature features of auxiliary subunits are their direct and stable interaction with principal subunits and effects on channel or receptor kinetics and/or pharmacology. Additionally, auxiliary subunits often promote surface expression of channels or receptors. KCTD8, -12, -12b, and -16 subunits are examples of GPCR-associated proteins that fulfill some of the criteria of auxiliary receptor subunits (5). It is already well established that the KCTDs directly interact with the principal receptor subunit GABA_B2_ and that they affect kinetic and pharmacological properties of the receptor response. Our experiments establish that KCTD12 stably interacts with GABA_B_ receptors throughout the lifecycle of the receptor, as expected for an auxiliary receptor subunit. In the biosynthetic pathway, assembly of KCTD12 with the receptor takes place as early as in the ER compartment. This association is maintained during receptor trafficking to the plasma membrane, agonist activation, and internalization. We found that assembly with KCTD12 stabilizes GABA_B_ receptors at the cell surface. This is also supported by an earlier study showing that overexpression of KCTD12 in cultured hippocampal neurons augments axonal surface targeting of GABA_B_ receptors (4). Our experiments indicate that the increase in surface receptors is the result of reduced constitutive receptor internalization. In this context, it is interesting to note that the C-terminal domain of GABA_B2_, which comprises the KCTD12-binding site, regulates the rate of constitutive internalization, presumably through the shielding of a dileucine signal in the C terminus of GABA_B1_ (35). It is conceivable that KCTD12 modulates the GABA_B1_/GABA_B2_ interface in a manner that reduces constitutive internalization. It is interesting that KCTD12 shortens the rise time and increases the magnitude of the receptor response while at the same time promoting desensitization. Assembly with KCTD12 may therefore provide the means to increase the temporal precision of GABA_B_ receptor signaling. An increase in cell surface stability of the receptors is also mediated by the sushi domains in the extracellular domain of GABA_B1a_ (36). This demonstrates that both intracellular and extracellular mechanisms have evolved to stabilize GABA_B_ receptors at the cell surface. It is intriguing that only KCTD12 influences GABA_B_ receptor surface expression, whereas the KCTD8, -12b, and -16 subunits do not. The reason for this is unknown. Because the amino acid sequence identity between the KCTD8, -12, -12b, and -16 proteins is only between 33 and 54%, it may be argued that constitutive internalization at the GABA_B1_/GABA_B2_ interface is influenced by sequence elements that are unique to KCTD12.

We found that knock-out or knockdown of KCTD12 in cultured hippocampal neurons, which express high amounts of KCTD12 protein (6), decreases surface GABA_B_ receptor expression and the peak amplitude of the baclofen-induced K^+^ current. Acute knockdown of KCTD12 had a more pronounced effect in reducing surface receptors than the constitutive loss of KCTD12 in knock-out mice. Acute and dynamic changes in KCTD12 expression may influence surface GABA_B_ receptor expression more strongly because of the lack of compensatory changes. Regulation of GABA_B_ receptors via KCTD12 may be of relevance to disease. A genome-wide association study found that a polymorphism in the promoter region of the *KCTD12* gene is associated with bipolar I disorder (37). Likewise, KCTD12 has been associated with depressive disorders (38) and schizophrenia (39). We speculate that KCTD12 is necessary for the precise timing of GABA_B_ receptor-mediated inhibitory effects on network activity (40), perturbation of which would manifest as psychiatric disorders.

## Footnotes

3 The abbreviations used are:

ER: endoplasmic reticulum
KCTD: K^+^ channel tetramerization domain-containing protein
GPCR: G-protein coupled receptor
BRET: bioluminescence resonance energy transfer
Rluc: *Renilla* luciferase
Endo H: endoglycosidase H
DIV: days *in vitro*
pF: picofarads
GB1: GABA_B1a_
GB2: GABA_B2_
GB1ASAA: Myc-GABA_B1_ASAA
GB2Y902A: GABA_B2_Y902A.
